# Entwicklung eines Anforderungsprofils für Betriebliche Gesundheitsmanager:innen

**DOI:** 10.1007/s11553-022-01009-0

**Published:** 2023-01-17

**Authors:** Sebastian Wedel, Eberhard Nöfer, Astrid Schütz

**Affiliations:** 1 Lehrstuhl für Persönlichkeitspsychologie und Psychologische Diagnostik, Kompetenzzentrum für Angewandte Personalpsychologie, Otto-Friedrich-Universität Bamberg, Markusplatz 3, 96045 Bamberg, Deutschland; 2 Hochschule Coburg, Friedrich-Streib Str. 2, 96450 Coburg, Deutschland

**Keywords:** Betriebliches Gesundheitsmanagement, Betriebliche Gesundheitsförderung, Anforderungsanalyse, Ausbildung, Personalauswahl, Occupational health, Workplace health management, Occupational health promotion, Training, Staff selection

## Abstract

**Hintergrund:**

Die Aufgaben Betrieblicher Gesundheitsmanager:innen sind vielfältig. Für die zielgerichtete Personalauswahl und anforderungsgerechte Ausbildung im betrieblichen Gesundheitsmanagement (BGM) fehlt bislang aber ein entsprechendes Anforderungsprofil.

**Ziel der Arbeit:**

Ziel dieser Arbeit war die Entwicklung eines Anforderungsprofils für Betriebliche Gesundheitsmanager:innen unter Einbeziehung von Subject Matter Experts.

**Material und Methoden:**

Das Anforderungsprofil wurde mithilfe der Task Analysis Tools (TAToo) [6] erstellt. Das 3‑stufige Vorgehen umfasste 21 semistrukturierte Interviews mit Stelleninhaber:innen und Führungskräften, zwei Workshops sowie eine Online-Befragung (*n* = 46) zur ökologischen Validierung der Ergebnisse. Auf Grundlage dieser Informationen wurde ein Anforderungsprofil für Betriebliche Gesundheitsmanager:innen erstellt.

**Ergebnisse:**

Die Aufgaben, Ziele und Schnittstellen von Betrieblichen Gesundheitsmanager:innen sind vielfältig. Fachwissen aus den Bereichen Gesundheitswissenschaften, Psychologie, Ergonomie, Arbeitswissenschaften und Betriebswirtschaftslehre sind für die Tätigkeit im BGM besonders wichtig. Aus dem Bereich der Methodenkompetenzen sind Netzwerkfähigkeit, systematisches Arbeiten, Präsentationsvermögen sowie Projektmanagement relevant. Als wichtige Soft-Skills gelten Vertrauenswürdigkeit, Leidenschaft für das Thema Gesundheit, Begeisterungsfähigkeit sowie Zuverlässigkeit und Teamfähigkeit. COVID-19 („coronavirus disease 2019“) scheint das Anforderungsprofil leicht verändert zu haben, sodass die Themen Digitalisierung, gesunde Führung, psychische Gesundheit, Gesundheit im Homeoffice und Pandemiemanagement an Bedeutung gewonnen haben.

**Diskussion:**

Limitationen hinsichtlich der Verallgemeinerbarkeit des Anforderungsprofils können sich aus der mangelnden Repräsentativität der Stichprobe ergeben. Arbeitssicherheit, Arbeitsmedizin, betriebliches Eingliederungsmanagement (BEM), Evaluation und Digitalisierung sollten bei der Ausbildung von Betrieblichen Gesundheitsmanager:innen sowie bei der Personalauswahl eine größere Rolle als bislang spielen.

## Abstract (en)

**Background:**

The tasks of occupational health managers (OHMs) are diverse. Currently there is no up-to-date job profile for OHMs that can be used for demand-oriented selection of personnel and the development of training curricula in workplace health management (WHM).

**Objectives:**

The aim of this study was to develop a job profile for OHMs involving subject-matter experts.

**Materials and methods:**

We conducted a job analysis with Task Analysis Tools (TAToo) [6]. The three-step approach comprised 21 semi-structured interviews with job holders and supervisors, two workshops, and an online survey (*n* = 46) to ecologically validate the results. On that basis, a job profile for OHMs was developed.

**Results:**

The tasks, targets, and interfaces of OHMs are diverse. Expertise in health sciences, psychology, ergonomics, and business administration is particularly important for their work. Regarding methodological skills, networking, systematic work, presentation and project management skills are relevant. Important soft skills are trustworthiness, passion for health, enthusiasm, reliability, and the readiness for teamwork. COVID-19 has slightly changed the job profile, so that digital skills, healthy leadership, mental health, healthy home-offices, and pandemic preparedness have become more important.

**Conclusions:**

Limitations with regard to the generalizability of the job profile may result from the fact that the analysis is not based on a representative sample. Expertise around occupational safety, occupational health, return-to-work, evaluation, and digitalization should play a greater role in the training and selection of OHMs.

Die Aufgaben Betrieblicher Gesundheitsmanager:innen sind vielfältig [8]. Für die anforderungsgerechte Ausbildung sowie zielgerichtete Personalauswahl fehlt bislang aber ein entsprechendes Anforderungsprofil, welches neben Zielen und Aufgaben der Tätigkeit auch Informationen zu relevanten Kenntnissen und Fähigkeiten enthält. In diesem Beitrag wird die Entwicklung eines solchen Anforderungsprofils für Betriebliche Gesundheitsmanager:innen beschrieben.

## Hintergrund und Fragestellung

Betriebliches Gesundheitsmanagement (BGM) gewinnt zunehmend an Bedeutung. Bereits im Jahr 2017 kam eine Studie zu dem Schluss, dass der Arbeitsmarkt für gut ausgebildete Betriebliche Gesundheitsmanager in Zukunft wachsen wird [9]. Darüber hinaus scheint das BGM durch die COVID-19-Pandemie („coronavirus disease 2019“) weiter an Bedeutung zu gewinnen [1]. Ernährung, Bewegung, Stressbewältigung, Wiedereingliederung und Arbeitsgestaltung sind nur einige Themenfelder, mit welchen sich Betriebliche Gesundheitsmanager:innen in ihrem beruflichen Alltag befassen [11]. Blickt man auf die Qualifikationen der in Organisationen beschäftigten Betrieblichen Gesundheitsmanager:innen wird deutlich, dass ihre Qualifikationsprofile sehr vielfältig sind. So finden sich in der Gruppe u. a. Gesundheitswissenschaftler:innen, Psycholog:innen, Mediziner:innen und Sportwissenschaftler:innen.[2]. Eine im Jahr 2021 durchgeführte Analyse der akademischen Ausbildungskonzepte zum BGM zeigte, dass sich die Ausbildungsinhalte von Studiengängen im BGM erheblich unterscheiden [12]. Hinzu kommt ein breiter Markt an nicht-akademischen Weiterbildungen im BGM, welcher durch die Bildungsempfehlungen und Zertifizierungen des Bundesverbandes Betriebliches Gesundheitsmanagement e. V. (BBGM) teilweise standardisiert ist [4].

Mit Blick auf die steigende Relevanz des BGM, die Unterschiedlichkeit der Qualifikationsprofile sowie die Heterogenität der Ausbildungslandschaft wird die Notwendigkeit eines allgemeinen Anforderungsprofils im BGM deutlich, an welchem sich Unternehmen bei der Personalauswahl sowie Bildungsträger bei der Entwicklung ihrer Curricula orientieren können. Im Rahmen dieser Studie wurde mithilfe von 21 Interviews, 2 Workshops und einer quantitativen Befragung (*n* = 46) untersucht, welche Aufgaben und Ziele Betriebliche Gesundheitsmanager:innen haben, und welches Wissen, welche Kenntnisse und welche Fähigkeiten sie benötigen, um den Anforderungen gerecht zu werden. Auf dieser Basis wurde ein ökologisch validiertes Anforderungsprofil für Betriebliche Gesundheitsmanager:innen erstellt.

## Studiendesign und Untersuchungsmethoden

Die Entwicklung des Anforderungsprofils erfolgte mithilfe der Task Analysis Tools (TAToo; [6]). Das gewählte Vorgehen umfasste die folgenden 3 Schritte:

- *Erheben* von Zielen, Aufgaben und Schnittstellen, erfolgsentscheidenden „knowledge, skills, abilities“ (KSA) sowie erfolgskritischen Verhaltensweisen von Betrieblichen Gesundheitsmanager:innen,
- *Gruppieren* der kritischen Verhaltensweisen zu verhaltensnahen Anforderungen,
- *Bewerten* der gesammelten KSA und verhaltensnahen Anforderungen hinsichtlich ihrer Bedeutsamkeit und ihrer Trainierbarkeit.

Bei der Erstellung des Anforderungsprofils wurden neben Stelleninhaber:innen auch Führungskräfte von Betrieblichen Gesundheitsmanager:innen einbezogen. Um ein möglichst vollständiges Anforderungsprofil zu erhalten, wurde bei der Auswahl der Teilnehmenden darauf geachtet, dass sie aus möglichst unterschiedlichen Unternehmen bzw. Einrichtungen kamen sowie unterschiedliche Ausbildungen und unterschiedlich lange Berufserfahrung hatten.

### Schritt 1: Erheben

Im Rahmen von Schritt 1 „Erheben“ wurden 21 semistrukturierte Interviews mit Stelleninhaber:innen und Führungskräften von Betrieblichen Gesundheitsmanager:innen durchgeführt, in welchen die Interviewpartner:innen zu den Zielen, Aufgaben, Schnittstellen sowie zur notwendigen Qualifikation und erfolgsentscheidenden KSA von Betrieblichen Gesundheitsmanager:innen befragt wurden. Entsprechend des von Koch u. Westhoff (2012) vorgeschlagenen Vorgehens [6] im Sinne der „critical incident technique“ [5] wurden zudem erfolgskritische Verhaltensweisen aus dem beruflichen Alltag von Betrieblichen Gesundheitsmanager:innen erhoben. Die Rekrutierung der Interviewpartner:innen erfolgte über das persönliche Netzwerk der Autor:innen. Voraussetzung für eine Ansprache war, dass die Personen als Betriebliche Gesundheitsmanager:innen bzw. als Führungskräfte von Betrieblichen Gesundheitsmanager:innen tätig waren. Zudem wurde darauf geachtet, dass die Personen unterschiedliche Ausbildungshintergründe und unterschiedlich lange Berufserfahrung aufwiesen sowie in Unternehmen/Einrichtungen unterschiedlicher Branche und Größe arbeiteten. Unter den 21 Interviewpartner:innen waren schließlich 18 Akademiker:innen und 3 Nichtakademiker:innen mit unterschiedlicher Berufserfahrung, aus Unternehmen verschiedener Größe und mit verschiedenen Ausbildungshintergründen, z. B. Gesundheitswissenschaftler:innen, Sportwissenschaftler:innen, ein Mediziner, ein Psychologe, eine Juristin, ein Physiotherapeut sowie Angehörige anderer Professionen.

Die Interviews wurden mithilfe von Videokonferenzen durchgeführt, dauerten zwischen 45 und 60 Minuten und wurden mit Einverständnis der Interviewpartner:innen aufgezeichnet. Die im Rahmen der Interviews erhobenen Informationen wurden während der Interviews stichpunktartig notiert und anschließend mithilfe der Interviewaufzeichnungen ergänzt. Alle Ergebnisse aus den Interviews wurden schließlich zusammengefasst und in ein vorläufiges Anforderungsprofil aufgenommen.

### Schritt 2: Gruppieren

Ziel von Schritt 2 „Gruppieren“ war es, die in den Interviews gesammelten erfolgskritischen Verhaltensweisen zu verhaltensnahen Anforderungen zusammenzufassen. Dafür wurden zwei aufeinander aufbauende, halbtägige Workshops mit 5 Stelleninhaber:innen und Führungskräften aus Schritt 1 sowie 3 Hochschulprofessor:innen aus den Gesundheitswissenschaften durchgeführt. Die Hochschulprofessor:innen wurden zusätzlich zu den Stelleninhaber:innen und Führungskräften einbezogen, um die akademische Perspektive hinsichtlich des BGM zu vertreten. In den Workshops wurden die in den Interviews genannten 228 kritischen Verhaltensweisen aus 42 kritischen Ereignissen in mehreren Teilschritten zu 26 verhaltensnahen Anforderungen zusammengefasst. Diese 26 verhaltensnahen Anforderungen wurden in der Folge in das vorläufige Anforderungsprofil aufgenommen.

### Schritt 3: Bewerten

**Abb. 1:**
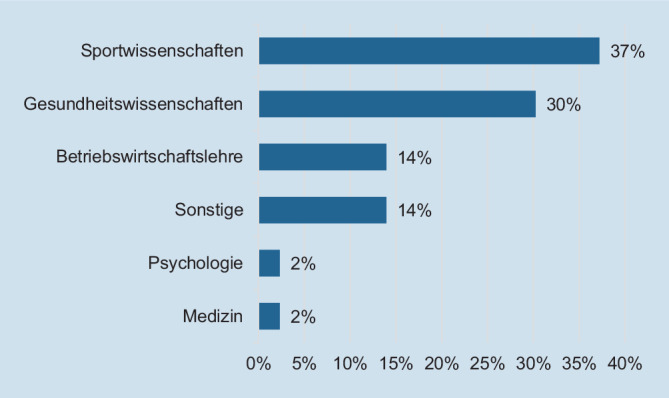
Verteilung der akademisch ausgebildeten Studienteilnehmer:innen nach Studienrichtung

Im Rahmen von Schritt 3 „Bewerten“ wurde das vorläufige Anforderungsprofil mittels Online-Befragung validiert. Zielgruppe der Online-Befragung waren, wie bereits bei den Interviews in Schritt 1, Stelleninhaber:innen und Führungskräfte von Betrieblichen Gesundheitsmanager:innen. Die Zugehörigkeit zu dieser Zielgruppe war das einzige formale Inklusionskriterium für die Teilnahme an der Online-Befragung. Die Zielgruppenansprache erfolgte zum einen über Business Social Media der Autor:innen, wodurch sich aufgrund des deutschsprachigen Einladungstextes und Fragebogens vermutlich ausschließlich deutschsprachige Stelleninhaber:innen und Führungskräfte angesprochen fühlten. Zusätzlich wurden die Interviewpartner:innen aus Schritt 1 zur Teilnahme an der Online-Befragung eingeladen und die Gruppe im Schneeballverfahren erweitert. Im Rahmen der Befragung wurden alle KSA und verhaltensnahen Anforderungen hinsichtlich ihrer Bedeutsamkeit und ihre Trainierbarkeit bewertet. Unter Bedeutsamkeit war in diesem Kontext die Relevanz des jeweiligen Aspekts für die berufliche Praxis im BGM zu verstehen („Wie bedeutsam finden Sie persönlich die folgenden Wissens- oder Themengebiete für die Erbringung einer guten Arbeitsleistung als Betriebliche/r Gesundheitsmanager/in?“). Bei der Trainierbarkeit ging es um die Frage, inwiefern sich die jeweiligen Kompetenzen z. B. mithilfe von Trainingsmaßnahmen erwerben lassen („Lassen sich Ihrer Erfahrung nach die folgenden Wissens- oder Themengebiete trainieren, also die jeweiligen Kompetenzen dafür erwerben [z. B. mithilfe von Trainingsmaßnahmen]?“). Alle im Rahmen der Interviews erhobenen KSA und verhaltensnahen Anforderungen, die im Rahmen der Online-Befragung mit einer Bedeutsamkeit von ≥ 3 im Median auf einer Skala von 1–5 (1 = gar nicht wichtig, 2 = wenig wichtig, 3 = mittelmäßig wichtig, 4 = ziemlich wichtig, 5 = sehr wichtig) bewertet wurden, wurden zusammen mit einer Information hinsichtlich ihrer Trainierbarkeit (1 = ja, 2 = eher ja, 3 = eher nein, 4 = nein) in das endgültige Anforderungsprofil aufgenommen. Insgesamt wurden 46 Datensätze für die Ergebnisaufbereitung berücksichtigt. Die durchschnittliche Berufserfahrung der Teilnehmenden an der Online-Befragung betrug 9 Jahre (Minimum [Min] = 0, Maximum [Max] = 30, Mean = 9,01, Standardabweichung [SD] = 7,5). Unter den Studienteilnehmer:innen befanden sich 33 Stelleninhaber:innen und 13 Führungskräfte, 43 Akademiker:innen und 3 Nichtakademiker:innen. Abb. 1 zeigt die Aufteilung der 43 akademisch ausgebildeten Teilnehmenden auf verschiedene Studienrichtungen.

Die meisten Studienteilnehmer:innen hatten eine sportwissenschaftliche oder gesundheitswissenschaftliche Ausbildung. „Sonstige“ Ausbildungen hatten eine juristische, erziehungswissenschaftliche, umweltwissenschaftliche, sozialpädagogische oder physiotherapeutische Ausrichtung. Die Verteilung der Teilnehmenden hinsichtlich der Größe der Unternehmen und Einrichtungen, in welchen sie beschäftigt waren, ist in Tab. 1 dargestellt. Ein Großteil der Teilnehmenden war zum Zeitpunkt der Befragung in Unternehmen mit mehr als 1000 Mitarbeitenden beschäftigt.

**Tab. 1:** Verteilung der Studienteilnehmer:innen nach Unternehmensgröße

Beschäftigte (*n*)	Häufigkeit (*n*)
0–50	6
51–250	0
251–1.000	4
1.001–10.000	14
> 10.000	22
Gesamt	46

**Abb. 2:**
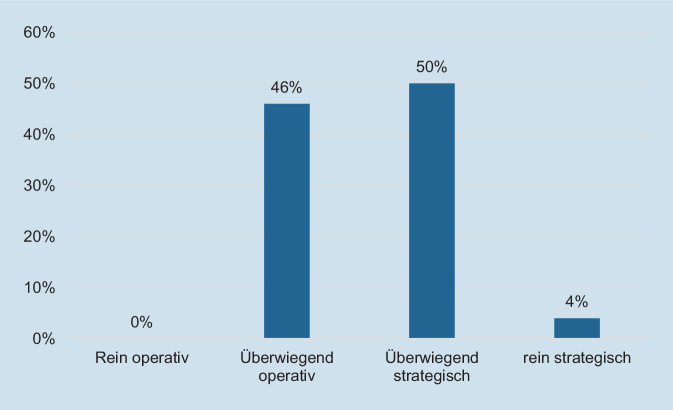
Verteilung nach Ausrichtung

Die Verteilung der Teilnehmenden nach der Ausrichtung ihrer Tätigkeit ist in Abb. 2 dargestellt. Demnach waren mit 54 % die leichte Mehrheit der Befragten eher strategisch tätig.

## Ergebnisse

Das Ergebnis des dreistufigen Vorgehens ist ein ökologisch validiertes Anforderungsprofil für Betriebliche Gesundheitsmanager:innen, welches für Personalplanung, Personalauswahl, Personalbeurteilung sowie für Trainings und Personalentwicklung im BGM verwendet werden kann [3, 7]. Wie oben bereits erwähnt, wurden alle Wissensgebiete, Kompetenzen bzw. verhaltensnahen Anforderungen, die in der Online-Befragung mit einem Medianwert ≥ 3 bewertet wurden, sowie eine Zusammenfassung der in den Interviews genannten Ziele, Aufgaben, Schnittstellen und Qualifikationen in das Anforderungsprofil aufgenommen.

Das Anforderungsprofil setzt sich aus 4 Teilen zusammen:

- Teil I: Beschreibung der Tätigkeit Betrieblicher Gesundheitsmanager:innen (aus Schritt 1),
- Teil II: Qualifikation, Wissen und Kenntnisse (aus Schritt 1 und 3),
- Teil III: verhaltensnahe Anforderungen (aus Schritt 1, 2 und 3),
- Teil IV: Veränderungen des Anforderungsprofils durch COVID-19 (aus Schritt 1 und 3).

### Teil I: Beschreibung der Tätigkeit Betrieblicher Gesundheitsmanager:innen

Die Ziele und Aufgaben Betrieblicher Gesundheitsmanager:innen sind vielfältig. Genannt wurden Themen wie der Erhalt der Arbeitsfähigkeit und Leistungsbereitschaft der Mitarbeitenden, die Förderung der Gesundheit von Mitarbeitenden, die Schaffung gesundheitsförderlicher Arbeitsbedingungen (Verhältnisprävention) sowie die Steigerung der Arbeitgeberattraktivität. Als Aufgaben wurden z. B. die strategische Entwicklung/Etablierung eines ganzheitlichen BGM-Systems, die Bedarfsermittlung zur Mitarbeitendengesundheit, die Planung und Umsetzung von Maßnahmen in den Bereichen gesunde Ernährung, Bewegung, psychosoziale Gesundheit, Arbeitsmedizin und gesunde Arbeitsumgebung und schließlich die Evaluation von Maßnahmen der betrieblichen Gesundheitsförderung genannt. Als Schnittstellen Betrieblicher Gesundheitsmanager:innen wurden eine Vielzahl interner sowie externer Stellen identifiziert. Als interne Schnittstellen erkannten die Interviewpartner:innen z. B. Betriebsleitungen, Betriebsärzte, Fachkräfte für Arbeitssicherheit, Personalabteilungen und betriebliche Interessenvertretungen. Als externe Schnittstellen waren z. B. Berufsgenossenschaften, Krankenkassen sowie externe Gremien wie die Bundesanstalt für Arbeitsschutz und Arbeitsmedizin oder die Initiative Neue Qualität der Arbeit (INQA) relevant.

### Teil II: Qualifikation, Wissen und Kenntnisse

Mit Blick auf die erforderliche Qualifikation Betrieblicher Gesundheitsmanager:innen gab die große Mehrheit der Interviewpartner:innen (85 %; *n* = 21) sowie der Teilnehmenden an der Online-Befragung (80 %; *n* = 46) an, dass ein abgeschlossenes Hochschulstudium wichtig oder sehr wichtig sei. Als relevante Studiengänge wurden grundständige Studiengänge mit Gesundheitsbezug, wie Ernährungswissenschaften, Gesundheitswissenschaften, Medizin, Psychologie und Sportwissenschaften genannt. Als postgraduale Studiengänge wurden Masterstudiengänge aus dem Bereich Betriebswirtschaftslehre oder Gesundheitsmanagement genannt. Auch nicht-akademischen Weiterbildungen im BGM wurde eine praktische Relevanz zugesprochen.

#### Fachwissen

Die Tab. 2 zeigt die 25 wichtigsten Fachkompetenzen absteigend nach ihrer Bedeutsamkeit. Demnach sind v. a. gesundheitswissenschaftliche Kenntnisse, sowie Fachwissen aus den Bereichen Psychologie, Ergonomie, Arbeitswissenschaften und Betriebswirtschaftslehre von Bedeutung. Insgesamt wurden 58 Themengebiete aus dem Bereich Fachwissen mit einem Medianwert von ≥ 3 bewertet und damit in das Anforderungsprofil aufgenommen. Darunter befanden sich auch die gemäß einschlägigen Modellen zum BGM [10] relevanten Disziplinen Arbeitssicherheit, Arbeitsmedizin (Arbeits- und Gesundheitsschutz) sowie das betriebliche Eingliederungsmanagement (BEM). Demnach sind das BEM mit einer durchschnittlichen Bewertung seiner Bedeutung von 3,76 (*n* = 45; Mean = 3,76; SD = 0,85; Min = 2; Max = 5), Arbeitssicherheit mit einer Bedeutsamkeitsbewertung von 3,57 (*n* = 46; Mean = 3,57; SD = 0,82; Min = 2; Max = 5) und Arbeitsmedizin mit 3,37 (*n* = 46; Mean = 3,37; SD = 0,79; Min = 2; Max = 5) für eine Tätigkeit als Betriebliche/r Gesundheitsmanager/in von praktischer Relevanz.

**Tab. 2:** TOP 25 – Fachwissen im betrieblichen Gesundheitsmanagement

Fachwissen	Bewertung – Ø	SD	Trainierbar (Median)
Gesundheitswissenschaften – Theorien und Konzepte im BGM/in der betrieblichen Gesundheitsförderung (Erfolgsfaktoren, Nachhaltigkeit, Implementierung, Vernetzung, Kennzahlen)	4,43	0,82	Ja
Gesundheitswissenschaften – Salutogenese/Gesundheitsförderung	4,39	0,64	Ja
Gesundheitswissenschaften – Prävention (Primär‑, Sekundär‑, Tertiärprävention; Verhältnis- und Verhaltensprävention)	4,37	0,82	Ja
Gesundheitswissenschaften – Gesundheitskommunikation	4,33	0,81	Ja
Gesundheitswissenschaften – Wechselwirkungen von Arbeit und Gesundheit	4,30	0,78	Ja
Gesundheitswissenschaften – Allgemein	4,13	0,77	Ja
Betriebswirtschaftslehre (BWL) – Personalmanagement (Personalentwicklung, Führung und Gesundheit etc.)	4,11	0,84	Eher ja
Gesundheitswissenschaften – Theorien und Konzepte zu Gesundheit	4,11	0,81	Ja
Ergonomie	4,04	0,69	Ja
Psychologie – positive Psychologie, Resilienz	4,04	0,75	Eher ja
Psychologie – Stressbewältigung und Entspannung	4,02	0,77	Eher ja
Gesundheitswissenschaften – Settingansätze	4,00	0,96	Ja
Psychologie – Gesundheitspsychologie	4,00	0,72	Eher ja
BWL – Marketing	3,93	1,05	Ja
Psychologie – Verhaltenspsychologie (Motivation, Gruppendynamiken)	3,93	0,70	Eher ja
Arbeitswissenschaften – Analysemethoden (z. B. Methoden zur Analyse arbeitsbedingter Belastungen)	3,91	0,86	Ja
Digitalisierung	3,87	0,77	Eher ja
Psychologie – Arbeits- und Organisationspsychologie	3,87	0,68	Eher ja
Arbeitswissenschaften – Arbeitsfähigkeit und Arbeitsunfähigkeit	3,76	0,91	Ja
BWL – strategisches Management	3,76	0,96	Eher ja
Betriebliches Eingliederungsmanagement (BEM)	3,76	0,85	Ja
Gesundheitswissenschaften – Public Health (z. B. Public Health Action Cycle)	3,72	0,97	Ja
Arbeitswissenschaften – Arbeitswelt 4.0	3,70	0,80	Ja
Arbeitswissenschaften – Alters- und alternsgerechtes Arbeiten	3,65	0,67	Ja
BWL – Kosten-Nutzen-Analysen	3,59	0,85	Ja

*SD* Standardabweichung, *BGM* betriebliches Gesundheitsmanangement

Alle Themengebiete aus dem Bereich Fachwissen sind mit einer Bewertung der Trainierbarkeit von „ja“ oder „eher ja“ relativ gut trainierbar.

#### Methodenwissen

Die Bewertung der Bedeutsamkeit und Trainierbarkeit der im BGM relevanten Methodenkompetenzen ist in Tab. 3 dargestellt. Hier werden die Bereiche Netzwerkfähigkeit, systematisches Arbeiten, Präsentation sowie Projektmanagement als besonders relevant genannt. Auch die Methodenkompetenzen werden als gut trainierbar eingeschätzt.

**Tab. 3:** TOP 10 – Methodenwissen

Methodenwissen	Bewertung – Ø	SD	Trainierbar (Median)
Netzwerkfähigkeit (die Fähigkeit, soziale Beziehungen mit anderen Personengruppen einzugehen, um diese erfolgreich im Beruf einzusetzen)	4,63	0,53	Eher ja
Systematisches Arbeiten	4,43	0,65	Eher ja
Präsentation	4,43	0,50	Eher ja
Projektmanagement	4,41	0,57	Eher ja
Gesprächsführung	4,39	0,53	Eher ja
Moderation	4,35	0,67	Eher ja
Evaluation (z. B. Zielevaluation, Prozessevaluation)	4,22	0,66	Ja
Verfahren zur Einbeziehung von Stakeholdern in der Entscheidungsfindung (z. B. Fokusgruppeninterviews, World Cafés)	4,20	0,80	Eher ja
Coaching/Beratung	4,02	0,68	Eher ja
Medienkompetenz (Intranet, Social Media etc.)	3,96	0,83	Eher ja

*SD* Standardabweichung

#### EDV-Kenntnisse

Die Tab. 4 zeigt absteigend nach ihrer Bedeutsamkeit im BGM notwendige Kenntnisse in elektronischer Datenverarbeitung (EDV-Kenntnisse). Hier spielen mit einer durchschnittlichen Bewertung von 4,57 auf der fünfstufigen Skala die Microsoft Office 365(Microsoft 2022, Redmond, WA, USA)-Anwendungen die mit Abstand wichtigste Rolle. Die genannten EDV-Kenntnisse werden ebenfalls als durchweg gut trainierbar eingeschätzt.

**Tab. 4:** EDV-Kenntnisse

EDV-Kenntnisse	Bewertung – Ø	SD	Trainierbar (Median)
Microsoft Office 365 (z. B. Word, Excel, PowerPoint, Forms)	4,57	0,68	Ja
Umfragesoftware (z. B. Microsoft Forms, SurveyMonkey)	3,74	0,76	Ja
Programme zur Foto- und Videobearbeitung	3,22	0,86	Ja
Statistikprogramme (z. B. SPSS, R)	3,11	0,91	Ja

*SD* Standardabweichung

#### Sonstige Merkmale

Bei den sonstigen Merkmalen bzw. den sog. Soft-Skills sind Vertrauenswürdigkeit, Leidenschaft für das Thema Gesundheit, Begeisterungsfähigkeit sowie Zuverlässigkeit und Teamfähigkeit besonders wichtig (Tab. 5). Es fällt auf, dass diese als sehr wichtig bewerteten Kenntnisbereiche als eher nicht trainierbar eingeschätzt werden.

**Tab. 5:** TOP 10 – sonstige Merkmale

Sonstige Merkmale	Bewertung – Ø	SD	Trainierbar (Median)
Vertrauenswürdigkeit	4,72	0,45	Eher nein
Leidenschaft für das Thema Gesundheit	4,70	0,55	Eher nein
Begeisterungsfähigkeit	4,67	0,47	Eher nein
Zuverlässigkeit	4,67	0,51	Eher nein
Teamfähigkeit	4,63	0,53	Eher nein
Empathie	4,54	0,58	Eher nein
Zielorientierung	4,54	0,54	Eher ja
Selbstbewusstsein/sicheres Auftreten	4,52	0,54	Eher ja
Fähigkeit zum Blick über den Tellerrand	4,50	0,58	Eher ja
Argumentationsfähigkeit	4,46	0,50	Eher ja

*SD* Standardabweichung

### Teil III: Verhaltensnahe Anforderungen

Die wichtigsten verhaltensnahen Anforderungen sind in Tab. 6 dargestellt. Hier sind Aspekte wie sichere und überzeugende Kommunikation, das Motivieren von Mitarbeitenden sowie Wissen um die Bedeutung von BGM zur Steigerung der Arbeitgeberattraktivität besonders bedeutsam.

**Tab. 6:** TOP 10 – verhaltensnahe Anforderungen

Ein/e Betriebliche/r Gesundheitsmanager/in …	Bewertung – Ø	SD	Trainierbar (Median)
Kommuniziert sicher und überzeugend auf allen Ebenen	4,65	0,48	Eher ja
Motiviert Mitarbeitende und Führungskräfte zur Teilnahme an Maßnahmen der betrieblichen Gesundheitsförderung	4,61	0,53	Eher ja
Kennt die Bedeutung von betrieblichem Gesundheitsmanagement zur Steigerung der Arbeitgeberattraktivität	4,59	0,57	Eher ja
Zeigt Eigeninitiative	4,59	0,53	Eher ja
Evaluiert Maßnahmen der betrieblichen Gesundheitsförderung	4,48	0,62	Eher ja
Arbeitet konstruktiv und lösungsorientiert mit Interessenvertretungen	4,48	0,54	Eher ja
Sieht Veränderungen als Chance	4,46	0,65	Eher nein
Hat Geduld, Ausdauer und Mut	4,43	0,58	Eher nein
Schafft niedrigschwellige Angebote im betrieblichen Gesundheitsmanagement	4,41	0,74	Eher ja
Verfügt über konzeptionelle Stärke	4,39	0,53	Eher ja

*SD* Standardabweichung

### Teil IV: Veränderungen des Anforderungsprofils durch COVID-19

Gegenstand der Untersuchung war auch die Frage, inwiefern sich das Anforderungsprofil von Betrieblichen Gesundheitsmanager:innen durch die COVID-19-Pandemie verändert hat. 71 % der Interviewpartner:innen (*n* = 21) und 62 % der Teilnehmenden an der Online-Befragung (*n* = 45) berichteten, dass sich das Anforderungsprofil durch die Pandemie verändert hat. Folgende Aspekte haben nach Einschätzung der Befragten an Bedeutung gewonnen:

- Digitalisierung des BGM,
- gesunde Führung,
- psychische Gesundheit,
- Gesundheit im Homeoffice,
- Pandemiemanagement.

Bei der Bewertung der Bedeutsamkeit aller in den Teilen II–IV genannten KSA bzw. verhaltensnahen Anforderungen wurden mit einer Ausnahme keine signifikanten Unterschiede zwischen Befragten aus verschiedenen Unternehmensgrößen bzw. Betrieblichen Gesundheitsmanager:innen mit eher operativem bzw. strategischen Fokus erkennbar. Einzig Fachwissen zum Thema „Arbeitsmedizin“ scheint für Betriebliche Gesundheitsmanager:innen in Großunternehmen > 10.000 Beschäftigten wichtiger zu sein als für Betriebliche Gesundheitsmanager:innen in Unternehmen ≤ 10.000 Beschäftigten (Student = 2,266; df = 44,0; *p* = 0,014; Cohens d = 0,669; *n* = 46).

#### Qualität und Stabilität des Anforderungsprofils

Durch die im Kapitel „Studiendesign und Untersuchungsmethoden“ angesprochene Auswahl heterogener Interviewpartner:innen sollte eine hohe Repräsentativität des Anforderungsprofils erreicht werden. Eine abschließende Bewertung des Anforderungsprofils im Rahmen der Online-Befragung hinsichtlich der Frage, wie gut die Anforderungen an Betriebliche Gesundheitsmanager:innen aus Sicht der Befragten im vorgelegten Anforderungsprofil abgebildet sind, ergab, dass das Anforderungsprofil zu 89 % die beruflichen Anforderungen im BGM abbildet (*n* = 44, Mean = 89 %, Min = 63, Max = 100, SD = 8,93). Somit kann das Anforderungsprofil für Betriebliche Gesundheitsmanager:innen als ökologisch valide betrachtet werden. Vergleicht man dieses Anforderungsprofil mit einem Anforderungsprofil aus dem Jahr 2012 (vgl. Strübin [2012] – unveröffentlichte Masterarbeit), welches mithilfe derselben wissenschaftlichen Methodik, jedoch einer kleineren Stichprobe, entwickelt wurde, so ergeben sich Hinweise auf Stabilität und Veränderung der notwendigen Kenntnisse und Fähigkeiten von Betrieblichen Gesundheitsmanager:innen. Demnach lassen sich deutliche Übereinstimmungen bei den als wichtig beschriebenen Fachkenntnissen (z. B. Gesundheitswissenschaften, Ergonomie, Psychologie), beim Methodenwissen (Präsentation, Projektmanagement, Gesprächsführung, Moderation, Evaluation) und bei den wichtigen EDV-Kenntnissen erkennen, sodass man davon ausgehen kann, dass die Basisanforderungen an Betriebliche Gesundheitsmanager:innen in den vergangenen 10 Jahren relativ stabil waren. Es lässt sich jedoch auch erkennen, dass der Anforderungskatalog umfangreicher wurde. So sind heute mit Personalmanagement, Gesundheitspsychologie, Sucht(prävention), Datenschutz und Fremdsprachenkenntnissen Themen wichtig, die vor 10 Jahren anscheinend noch nicht relevant waren. Zudem sind die bereits beschriebenen Veränderungen des Anforderungsprofils durch die COVID-19-Pandemie zu berücksichtigen, welche Hinweise auf spezifische Anforderungen in bzw. nach einer globalen Gesundheitskrise geben.

Das vollständige Anforderungsprofil kann hier abgerufen werden: https://osf.io/keacy

## Diskussion

Obwohl die Studienteilnehmer:innen die Stelle eines Betrieblichen Gesundheitsmanagers bzw. einer Betrieblichen Gesundheitsmanagerin in diesem Anforderungsprofil als sehr gut abgebildet sahen, könnte es dennoch Verzerrungen geben, die die Validität des Anforderungsprofils beeinflussen. Zwar wurde, um die Ergebnisqualität positiv zu beeinflussen, bei jedem Entwicklungsschritt auf eine breite Verteilung der Studienteilnehmer:innen hinsichtlich ihrer Ausbildungshintergründe geachtet. Dennoch war die Stichprobe nicht repräsentativ, sodass die Ergebnisse nicht zwingend verallgemeinerbar sind. Zudem war ein Großteil der Studienteilnehmer:innen in Schritt 3 zum Zeitpunkt der Befragung in Unternehmen mit mehr als 1000 Mitarbeitenden beschäftigt, sodass das Anforderungsprofil von Betrieblichen Gesundheitsmanager:innen in Unternehmen bis 1000 Mitarbeitenden vom hier erarbeiteten Anforderungsprofil abweichen könnte.

Darüber hinaus kann es in verschiedenen Unternehmen durchaus Unterschiede hinsichtlich des Anforderungsprofils der Betrieblichen Gesundheitsmanager:innen geben, z. B. wenn es im jeweiligen Unternehmen andere Stellen gibt, die für Arbeitssicherheit, Arbeitsmedizin oder BEM zuständig sind (z. B. Betriebsärzte, Fachkräfte für Arbeitssicherheit oder mit dem BEM beauftragte Personalmanager:innen). In solchen Fällen würde es vermutlich genügen, wenn Betriebliche Gesundheitsmanager:innen über ein Grundverständnis aus diesen Fachrichtungen verfügen.

Neben den verschiedenen Wissensgebieten, die für eine Tätigkeit als Betriebliche/r Gesundheitsmanager/in relevant sind, beinhaltet dieses Anforderungsprofil auch eine Reihe von Methodenkenntnissen, EDV-Kenntnissen, Soft-Skills sowie Persönlichkeitsmerkmalen, die im BGM relevant sind, wie z. B. Präsentation, Projektmanagement, EDV-Kenntnisse, Vertrauenswürdigkeit, Zuverlässigkeit etc. Auch in diesen Kompetenzbereichen können die relevanten Anforderungen von Unternehmen zu Unternehmen verschieden sein. Vor diesem Hintergrund erscheint es wichtig, dass das hier vorliegende Anforderungsprofil für die konkrete Tätigkeit in verschiedenen Unternehmen angepasst wird und die jeweils relevanten Wissensgebiete und Kompetenzen bei der Personalauswahl berücksichtigt werden. Für die Personalauswahl besonders relevant sind dabei die Anforderungen, die als eher nicht trainierbar eingestuft wurden, wie z. B. die Soft-Skills Vertrauenswürdigkeit, Leidenschaft für das Thema Gesundheit, Begeisterungsfähigkeit, Zuverlässigkeit und Teamfähigkeit.

Mit Blick auf Veränderungen des Anforderungsprofils durch die COVID-19-Pandemie bleibt abzuwarten, ob sich der temporäre Bedeutungszuwachs der Themen Digitalisierung, gesunde Führung, psychische Gesundheit, Gesundheit im Homeoffice und Pandemiemanagement auch nachhaltig im Anforderungsprofil niederschlägt oder ob diese Themen wieder an Bedeutung verlieren werden.

Wie oben bereits beschrieben sind die drei Handlungsfelder Betriebliche Gesundheitsförderung (BGF), der sich aus den Disziplinen Arbeitssicherheit und Arbeitsmedizin ergebende Arbeits- und Gesundheitsschutz (AGS), sowie das BEM entsprechend der einschlägigen Literatur wichtige Themen im BGM [10]. Eine Analyse der akademischen Ausbildungskonzepte im BGM [12] zeigte jedoch, dass sich Lerninhalte zum AGS sowie zum BEM mit ca. 2 % der Lerninhalte aller analysierten Studiengänge nur selten in den akademischen Ausbildungscurricula wiederfinden. Blickt man auf das hier vorliegende Anforderungsprofil und auf die insgesamt 58 aufgenommenen Fachkenntnisse, ist Wissen aus AGS und BEM im Einklang mit der oben genannten Literatur für den beruflichen Alltag von Betrieblichen Gesundheitsmanager:innen tatsächlich relevant. Neben AGS und BEM zeigt sich eine weitere Diskrepanz zwischen akademischen Ausbildungskonzepten und Anforderungsprofil im BGM für die Themen Evaluation und Digitalisierung. Auch hier ergab die oben angesprochene Analyse der akademischen Ausbildungskonzepte im BGM [12], dass diese Themen nur selten in Hochschulcurricula zu finden sind, obwohl sie laut des hier vorliegenden Anforderungsprofils wichtige Fach- bzw. Methodenkenntnisse darstellen. Nachdem all diese Wissensgebiete neben ihrer hohen praktischen Relevanz auch als gut trainierbar eingestuft wurden, sollten sie auch in akademischen Ausbildungscurricula mehr Berücksichtigung finden.

Dieses auf Basis eines empirischen Vorgehens entwickelte Anforderungsprofil ist ein großer Schritt hin zu einer weiteren Professionalisierung des BGM. Es kann als wichtiges Bindeglied zwischen Theorie und Praxis betrachtet und zum Zwecke der Personalplanung, Personalauswahl, Personalbeurteilung sowie zur Trainings- und Personalentwicklung Anwendung finden. Damit ist es für die Praxis hoch relevant.

## Fazit für die Praxis

- Das Anforderungsprofil von Betrieblichen Gesundheitsmanager:innen umfasst eine Vielzahl verschiedenster Fach‑, Methoden‑, Selbst- und EDV-Kompetenzen (elektronische Datenverarbeitung).
- Expertise in den Bereichen Arbeits- und Gesundheitsschutz (AGS), betriebliches Eingliederungsmanagement (BEM), Digitalisierung und Evaluation ist wichtig für die Tätigkeit als Betriebliche:r Gesundheitsmanager:in. Dies sollte bei der Entwicklung von Ausbildungscurricula und bei der Personalauswahl berücksichtigt werden.
- Je nach Unternehmen können die an Betriebliche Gesundheitsmanager:innen gestellten Anforderungen von diesem Anforderungsprofil abweichen.
- Akademische Ausbildungscurricula im betrieblichen Gesundheitsmanagement (BGM) sollten vor dem Hintergrund dieses Anforderungsprofils hinsichtlich ihrer Lerninhalte überprüft und ggf. angepasst werden.
